# Trend of minimally processed and ultra-processed beverages purchased in Brazilian households: Less milk and much soft drink (2002–2003 to 2017–2018)

**DOI:** 10.3389/fpubh.2022.956142

**Published:** 2022-11-03

**Authors:** Natália Oliveira, Daniela Silva Canella

**Affiliations:** ^1^Postgraduate Program in Food, Nutrition and Health, Rio de Janeiro State University, Rio de Janeiro, Brazil; ^2^Institute of Nutrition, Rio de Janeiro State University, Rio de Janeiro, Brazil

**Keywords:** sugar-sweetened beverages, artificially sweetened beverages, ultra-processed foods, ultra-processed beverages, population health, diet quality, trends

## Abstract

The consumption of ultra-processed beverages, including sugar-sweetened and artificially sweetened ones, is associated with several health problems, which is different considering minimally processed beverages. The objective of this study was to assess the trends in the volume of minimally and ultra-processed beverages purchased for consumption in Brazilian households and their relationship with the proportion of dietary energy derived from ultra-processed foods and beverages. Drawing on data from the nationwide 2002–03, 2008–09, and 2017–18 Household Budget Surveys, the daily volume of beverages purchased *per capita* (milligrams) was investigated. The minimally processed beverages purchased declined over the period [2002–2003: $\bar{x}$ 156.5 ml (95%CI: 148.3–164.8); 2017–2018: $\bar{x}$ 101.6 ml (95%CI: 98.1–105.1)] and ultra-processed beverages were stable [2002–03: $\bar{x}$ 117.9 ml (95%CI: 108.1–127.7); 2017–18: $\bar{x}$ 122.8 (95%CI: 111.2–134.4)]. The most purchased beverage in 2002–2003 was milk [$\bar{x}$: 154.7 ml (95%CI: 146.4–162.9)], while in 2017–2018 regular soft drinks were the most purchased [$\bar{x}$: 110.7 ml (95%CI: 99.2–122.2)]. There was a decrease in the purchase of whole and skimmed milk and an increase in the purchase of other ultra-processed beverages between the periods. With the increase in the proportion of ultra-processed foods and beverages in the diet, the volume of ultra-processed beverage purchases rose and minimally processed beverages declined. The monitoring of beverage consumption and the implementation of public policies, such as taxation on ultra-processed beverages, are essential to promote improvements in health and curbing non-communicable diseases.

## Introduction

Beverage consumption, considering alcoholic or non-alcoholic and caloric or non-caloric ones, can be considered a topic of interest to public health due to their association with health outcomes. Among non-alcoholic beverages, there are minimally processed beverages, such as milk, water, and natural juices, and ultra-processed beverages, such as sugar- and artificially sweetened beverages (1). Sweetened beverages include sugar and artificially sweetened beverages (2). Sugar-sweetened beverages (SSB) are defined as all types of beverages that contain free sugars (e.g., carbonated soft drinks; non-carbonated drinks; artificial juices; liquid and powder concentrates; ready-to-drink tea; flavored dairy drinks, and others) (2, 3), while artificially sweetened beverages are those that use artificial sweeteners in their composition to replace sugar (4, 5).

Consumption of sweetened beverages, especially SSB, is associated with weight gain (6, 7), type 2 diabetes (8), high blood pressure (9), and obesity-related cancers (4, 10). Furthermore, evidence suggests that the consumption of artificially sweetened beverages is associated with cancers not related to obesity (11) since their (re)formulation tends to increase the use of food additives (5, 12), which in turn are related to intestinal dysbiosis, colitis, and metabolic syndrome (13, 14). This adverse relationship is not observed for minimally processed beverages, such as milk (1, 15).

SSB is one of the main sources of sugar in the Brazilian diet (16–18). In 2008–2009, the average energy share of added sugar was around 16%, a value that exceeds by almost 60% of the maximum consumption limit recommended by the World Health Organization (19). It is noteworthy that in 1987–1988, the fraction of household availability of sugar from soft drinks represented 5.6%, and in 2008–2009, it increased to 15.5% (18).

Although the proportion of energy in the diet derived from ultra-processed foods and beverages, henceforth called ultra-processed foods (UPF), has increased in Brazil and in 2017–2018, soft drinks represent one of the food items with the highest average daily consumption per capita (67.1 g/day) (20), as a whole, there has been a general decline in the rate of regular consumption (5 days/week or much) of sweetened beverages in the major Brazilian cities (21, 22). Typically, the energy of UPF is used to determine their dietary contribution and, consequently, diet/light beverages are overlooked in these analyses. However, studies have shown that artificially sweetened beverages are also associated with unfavorable health outcomes (11, 13, 14).

Sweetened beverage consumption in Brazil is subject to monitoring since a reduction in routine consumption is one of the targets defined in the national initiative *Strategic Actions Plan for Tackling Chronic Non-Communicable Diseases 2021–2030* (23). Considering the trend of worsening diet quality observed in Brazil and the potential replacement in the kind of beverages consumed, the objective of the present study was to assess the trends in the volume of minimally and ultra-processed beverages purchased for consumption in Brazilian households during the periods 2002–2003 and 2017–2018, and their relationship with the proportion of dietary energy derived from UPF.

## Materials and methods

The data used were drawn from the nationwide Household Budget Surveys (HBS) conducted by the Brazilian Institute of Geography and Statistics (IBGE) in 2002–2003, 2008–2009, and 2017–2018 with the Brazilian population. HBS has a representative sample of the Brazilian population and employs a complex clustered sampling procedure, involving geographic and socioeconomic stratification of all census tracts of the country, followed by random selection of tracts in the first stage and of households in the second stage. HBS assesses the structures of consumption, expenditures, income, and the variation in assets of families, offering a profile of the population's living conditions based on the analysis of household budgets. The total sample of households surveyed was 48,470 in 2002–2003, 55,970 in 2008–2009, and 57,920 in 2017–2018, distributed into 443, 550, and 575 sample strata, respectively, homogenous for socioeconomic status and geographic location.

Data collection from all surveys was carried out over 12 months, evenly distributed across strata, ensuring representativeness in the four trimesters of the year. These procedures are detailed in specific publications (20). All data are in the public domain and are available online on the IBGE website (https://www.ibge.gov.br/estatisticas/sociais/populacao/24786-pesquisa-de-orcamentos-familiares-2.html?=&t=microdados). Information contained in the database is confidential, excluding specific data about each family, such as individual identification, address, and telephone number.

Information on the purchases of food and beverage items for home consumption over a period of seven consecutive days was recorded daily by dwellers of the household or by the interviewer in a shared logbook of all purchases made. Due to the relatively short data collection period, sample strata were employed as the unit of study, providing a more accurate picture of annual food purchases. At the strata level, extreme differences between households are attenuated (considering, e.g., households that had no purchases during the 7 days of collection or those that made purchases for the entire month, behaviors characteristic of Brazilian families). Data referring to income and the number of dwellers in the households were also used to calculate the household *per capita* income.

The total amount of each food and beverage item purchased were converted to express daily grams or volume purchased per individual (grams or milliliters/*per capita*/day), after the exclusion of non-edible parts and the dilution of powdered beverages. All items were divided into four groups and subgroups of the NOVA classification system (1), with subsequent selection of subgroups of beverages (food group of interest in the present study). As these are acquisition data, natural juices were not evaluated because the fruit is usually acquired to make the juice.

Beverages were grouped into minimally processed beverages (whole milk, including fluid and diluted powdered; skimmed milk, including fluid and diluted powdered; and natural yogurts) and ultra-processed beverages [regular soft drinks; diet/light soft drinks; and other drinks (including dairy drinks without yogurt; regular artificial juices or teas; regular and diet/light soymilk powder; diet/light yogurts; diet/light milk-based drinks; and diet/light artificial juices and teas)].

The total daily *per capita* amount of each food and beverage item purchased was converted into energy (kcal), using the Brazilian Food Composition Table (TBCA) (24), and then the proportion of energy derived from the UPF group (including foods and beverages) was calculated.

Additionally, households were stratified according to the income level considering quintiles of *per capita* income and earning of at least the minimum wage, based on the values of January 2003 (R$ 240), 2009 (R$ 465), and 2018 (R$ 954).

Descriptive analyses were carried out through the means and respective 95% confidence intervals (95% CI) of volume (milliliters/*per capita*/day) of minimally processed and ultra-processed beverages for Brazil overall, and according to quintiles of income and earnings, the minimum wage for the three periods was surveyed (2002–2003; 2008–2009; and 2017–2018). Statistical significance was tested for the variations using linear regression models for the analysis of the temporal trend of estimates. Considering the relationship between food consumption and income (25, 26), based on linear regression models, the expected values (values predicted by the model) were calculated for the means adjusted by income (as a continuous variable). Therefore, means (obtained in the predicted model) and respective 95% CI of volume for all subgroups of minimally processed and ultra-processed beverages were described for the three periods surveyed. The same descriptive analyses were performed according to quartiles of the proportion of energy derived from UPF.

Based on the comparison of 95% CI, significant differences between the survey periods were evident. A lack of overlap between the intervals was assumed as a significant difference, adopting a significance level of 5%.

All analyses were carried out using the statistics package Stata/SE version 16.0 (Stata Corp., College Station, USA) on the *survey* module, which considers the effects of complex sampling and enables the extrapolation of results for the Brazilian population.

## Results

The trend of minimally processed and ultra-processed beverages purchased and their relationship with income is presented in Table 1. The volume of minimally processed beverages purchased declined over the period (*p-*trend = 0.000), while ultra-processed beverages remained stable (*p*-trend = 0.553) (Table 1).

**Table 1:** Mean and 95% confidence interval of daily *per capita* purchase of minimally processed and ultra-processed beverages according to income level.

**Income level**	**2002–03**	**2008–09**	**2017–18**	***p*-trend**
	**$\bar{x}$ ml (95%CI)**	**$\bar{x}$ ml (95%CI)**	**$\bar{x}$ ml (95%CI)**	
**MINIMALLY PROCESSED BEVERAGES**				
Mean	154.79 (146.03–163.54)	122.01 (116.78–127.24)^c^	105.97 (102.29–109.64)^c^^,^ ^d^	0.000
**Quintiles of income**				
Q1	111.89 (101.77–122.01)	92.23 (84.82–99.65)^c^	87.57 (80.06–95.08)^c^^,^ ^d^	0.000
Q2	159.73 (135.72–183.73)	109.05 (98.37–119.73)^c^	99.85 (93.95–105.75)^d^	0.000
Q3	151.75 (133.73–169–78)	129.25 (116.32–142.19)	108.52 (100.07–116.98)^d^	0.000
Q4	156.50 (139.59–173.42)	144.15 (130.96–157.34)	114.95 (105.51–124.38)^c^^,^ ^d^	0.000
Q5	194.28 (178.44–210.12)	136.94 (127.32–146.56)^c^	119.03 (111.46–126.59)^c^^,^ ^d^	0.000
**Earning at least the minimum wage**				
No^a^	110.95 (102.03–119.86)	94.84 (86.20–103.47)	88.86 (80.22–97.50)^d^	0.001
Yes^b^	167.97 (158.02–177.92)	130.00 (123.93–136.08)^c^	109.33 (105.38–113.28)^c^^,^ ^d^	0.000
**ULTRA-PROCESSED BEVERAGES**				
Mean	113.82 (101.36–126.29)	117.39 (108.80–125.98)	131.73 (120.14–143.32)	0.553
**Quintiles of income**				
Q1	46.51 (33.77–59.24)	53.20 (43.65–62.77)	91.50 (74.95–108.05)^c^^,^ ^d^	0.000
Q2	80.56 (60.43–100.69)	101.99 (82.91–121.08)	131.78 (100.04–163.53)	0.010
Q3	95.49 (82.56–108.42)	107.16 (96.41–117.90)	116.00 (90.30–141.71)	0.201
Q4	138.01 (115.08–160.95)	143.04 (127.70–158.39)	151.38 (125.25–177.51)	0.441
Q5	211.70 (186.12–237.28)	185.55 (164.18–206.93)	168.35 (145.91–190.79)	0.014
**Earning at least the minimum wage**				
No^a^	47.10 (35.93–58.25)	58.27 (49.12–67.42)	92.85 (73.62–112.07)^c^^,^ ^d^	0.000
Yes^b^	133.90 (119.61–148.18)	134.79 (124.62–144.97)	139.37 (126.25–152.50)	0.549

Brazilian Household Budget Surveys, 2002–2003; 2008–2009; 2017–2018.

a number of strata in 2002–2003 = 125, 2008–2009 = 199; 2017–2018 = 161;

b number of strata in 2002–2003 = 318, 2008–2009 = 351, 2017–2018 = 414;

c significant difference compared to the previous year, based on lack of overlap between the intervals;

d significant difference between the years: 2002–2003 and 2017–2018, based on lack of overlap between the intervals.

The acquisition of both minimally processed and ultra-processed beverages was influenced by income in all analyzed periods, but the intensity and even the direction of changes over the years were different among income groups. For minimally processed beverages, among households in the first quintile of income level and earning less than one minimum wage, the decrease was slighter than among those wealthier. Furthermore, for Q1, Q2, and Q3 income levels, the acquisition decreased between 2002–2003 and 2008–2009 and remained stable in 2017–2018, while for Q4 and Q5, the average was maintained between 2002–2003 and 2008–2009 and only decreased from 2008–2009 to 2017–2018. For ultra-processed beverages, despite the general stability, households in the first quintile of income level and earning less than one minimum wage almost doubled the acquisition over the years, with a stable volume between 2002–2003 and 2008–2009 and an increase from 2008–2009 to 2017–2018. On the other hand, households in the fifth quintile presented a significant decrease in the volume purchased (Table 1).

Predicted values presented the same trend for minimally processed beverages [2002–2003: $\bar{x}$ 156.5 ml (95%CI: 148.3–164.8); 2008–09: $\bar{x}$ 116.2 ml (95%CI: 111.6–120.8); 2017–18: $\overset{\bar{\;}}{x}$ 101.6 ml (95%CI: 98.1–105.1)] and ultra-processed beverages [2002–2003: $\bar{x}$ 117.9 ml (95%CI: 108.1–127.7); 2008–2009: $\bar{x}$ 105.8 ml (95%CI: 99.2–112.5); 2017–2018: $\bar{x}$ 122.8 (95%CI: 111.2–134.4)]. Milk was the most purchased beverage in volume by Brazilians in 2002–2003 [$\bar{x}$ 154.7 ml (95%CI: 146.4–162.9)], while regular soft drinks were the most purchased in the last period surveyed [2017–2018: $\bar{x}$ 110.7 ml (95%CI: 99.2–122.2)]. There was a significant reduction in the purchase of milk [2002–2003: $\bar{x}$ 154.7 ml (95%CI: 146.4–162.9); 2008–2009: $\bar{x}$ 105.2 ml (95%CI: 100.7–109.8) and 2017–2018: $\bar{x}$ 94.5 ml (95%CI: 91.1–98.0)] and skimmed milk [2008–2009: $\bar{x}$ 10.9 ml (95% CI: 9.9–11.9) and 2017–18: $\bar{x}$ 7.0 ml (95%CI: 6.3–7.0)]. For ultra-processed beverages, there was a significant increase in the purchase of other drinks between 2002–2003 and 2017–2018 [$\bar{x}$ 8.6 ml (95%CI: 6.9–10.3); $\bar{x}$ 11.1 ml (95%CI: 10.4–11.8), respectively] (Table 2).

**Table 2:** Crude and predicted mean and 95% confidence interval of daily *per capita* purchase of minimally processed and ultra-processed beverages.

**Beverages**	**2002–03**		**2008–09**		**2017–18**	
	**Crude**	**Predicted^a^**	**Crude**	**Predicted ^a^**	**Crude**	**Predicted^a^**
	$\bar{x}$ **ml (95%CI)**	$\bar{x}$ **ml (95%CI)**	$\bar{x}$ **ml (95%CI)**	$\bar{x}$ **ml (95%CI)**	$\bar{x}$ **ml (95%CI)**	
**Minimally processed beverages**	154.79 (146.03–163.54)	156.52 (148.27–164.78)	122.01 (116.78–127.24)^c^	116.20 (111.62–120.77)^c^	105.97 (102.29–109.64)^c^^,^ ^d^	101.62 (98.11–105.13)^c^^,^ ^d^
Whole milk	153.21 (144.49–161.92)	154.65 (146.37–162.92)	109.40 (104.40–114.40)^c^	105.23 (100.68–109–78)^c^	97.23 (93.75–100.71)^c^^,^ ^d^	94.53 (91.10–97.97)^c^^,^ ^d^
Skimmed milk	–	–	12.52 (10.90–14.14)	10.89 (9.85–11.92)	8.65 (7.42–9.88)^c^	7.02 (6.33–7.71)^c^
Natural yogurt	0.04 (0.01–0.06)	0.05 (0.01–0.08)	0.08 (0.06–0.11)	0.08 (0.05–0.10)	0.08 (0.05–0.10)	0.07 (0.05–0.09)
**Ultra-processed beverages**	113.82 (101.36–126.29)	117.90 (108.13–127.66)	117.39 (108.80–125.98)	105.82 (99.15–112.52)	131.73 (120.14–143.32)	122.80 (111.22–134.37)
Regular soft drink	106.27 (94.53–118.01)	109.32 (99.87–118.77)	102.70 (95.09–110.30)	93.48 (87.08–99.88)	116.50 (105.31–127.68)	110.73 (99.23–122.24)
Diet/light soft drink	–	–	3.48 (2.70–4.26)	2.82 (2.30–3.34)	1.32 (0.96–1.68)^c^	0.93 (0.71–1.15)^c^
Other drinks^b^	7.55 (6.11–9.01)	8.58 (6.88–10.28)	11.22 (9.81–12.62)^c^	9.51 (8.60–10.44)	13.92 (12.55–15.28)^d^	11.13 (10.43–11.83)^d^

Brazilian Household Budget Surveys, 2002**–**2003; 2008**–**2009; 2017**–**2018.

a Mean based on linear regression models the expected values for income control (values predicted by model);

b in 2002**–**2003, included dairy drinks without yogurt; regular artificial juices or teas; regular and diet/light soymilk powder; in 2008**–**2009 and 2017**–**2018, included dairy drinks without yogurt; regular artificial juices or teas; regular and diet/light soymilk powder; diet/light yogurts; diet/light milk-based drinks; and diet/light artificial juices and teas;

c significant difference compared to the previous year, based on lack of overlap between the intervals;

d significant difference between the years: 2002**–**2003 and 2017**–**2018, based on lack of overlap between the intervals.

Overall, for all years surveyed, there was a decrease in the volume of minimally processed beverages and an increase of ultra-processed beverages purchased proportionally with the increase in the share of UPF in the diet, without significant difference. For regular soft drinks, the values for the fourth quartile almost doubled relative to the first one in 2008–2009 and 2017–2018 [Q1: $\bar{x}$: 67.9 ml (95%CI: 51.6–84.3); Q4: $\bar{x}$ 138.2 ml (95%CI: 112.9–163.6); Q1: $\bar{x}$ 63.6 ml (95%CI: 45.6–81.7); Q4: $\bar{x}$ 124.7 ml (95%CI: 97.9–151.5), respectively]. In the last period, there is a difference for diet/light soft drinks [2017–2018: Q1: $\bar{x}$ 0.5 ml (95%CI: 0.3–0.8); Q4: $\bar{x}$ 1.9 g/ml (95%CI: 0.8–3.0)] and others drinks [2017-18: Q1: $\bar{x}$ 6.2 g/ml (95%CI: 5.4–7.0); Q4: $\bar{x}$ 19.6 g/ml (95%CI: 16.6–22.5)] (Table 3).

**Table 3:** Predicted mean and 95% confidence interval of daily *per capita* purchase of minimally processed and ultra-processed beverages according to quartiles of proportion of energy derived from ultra-processed foods and beverages.

**Quartiles of UPF**	**2002–03**	**2008–09**	**2017–18**
	**$\bar{x}$ ml (95%CI)**	**$\bar{x}$ ml (95%CI)**	**$\bar{x}$ ml (95%CI)**
**MINIMALLY PROCESSED BEVERAGES**			
Q1	169.88 (149.37–190.38)	128.87 (115.07–142.66)	108.85 (100.44–117.27)^d^
Q2	161.94 (141.41–182.46)	109.32 (99.44–119.20)^c^	98.99 (92.07–105.92)^d^
Q3	143.63 (127.53–159.73)	105.91 (94.32–117.50)^c^	95.34 (87.20–103.49)^d^
Q4	148.88 (132.44–165.31)	113.98 (99.53–128.43)^c^	98.07 (87.70–108.45)^d^
**Whole milk**			
Q1	168.51 (147.96–189.05)	119.88 (105.74–134.03)^c^	103.74 (95.36–112.11)^d^
Q2	160.09 (139.57–180.62)	98.00 (88.36–107.65)^c^	91.58 (84.65–98.50)^d^
Q3	141.55 (125.28–157.82)	95.28 (83.91–106.65)^c^	89.09 (81.24–96.94)^d^
Q4	146.60 (130.15–163.04)	99.71 (85.27–114.14)^c^	86.24 (76.13–96.34)^d^
**Skimmed milk**			
Q1	-	8.91 (7.46–10.36)	5.07 (4.21–5.94)^c^
Q2	-	11.26 (9.61–12.90)	7.35 (6.24–8.45)^c^
Q3	-	10.53 (8.29–12.77)	6.16 (4.69–7.63)^c^
Q4	-	14.19 (9.84–18.54)	11.74 (8.77–14.70)
**Natural yogurts**			
Q1	0.06 (−0.01–0.13)	0.07 (−0.00–0.14)	0.04 (0.01–0.07)
Q2	0.07 (−0.02–0.15)	0.06 (0.02–0.10)	0.07 (0.04–0.10)
Q3	0.04 (0.01–0.07)	0.09 (0.02–0.17)	0.09 (0.03–0.15)
Q4	0.02 (−0.00–0.06)	0.08 (0.01–0.16)	0.10 (0.03–016)
**ULTRA-PROCESSED BEVERAGES**			
Q1	73.75 (57.25–90.25)	71.90 (53.72–90.08)	101.67 (84.32–119.02)
Q2	116.48 (96.86–136.10)	101.32 (89.59–113.05)	145.47 (117.60–173.35)^c^^,^ ^d^
Q3	132.54 (105.82–159.25)	129.19 (106.54–151.85)	119.77 (89.61–149.93)
Q4	152.43 (126.13–178.73)	147.02 (118.82–175.23)	133.46 (106.88–160.05)
**Regular soft drinks**			
Q1	67.94 (51.57–84.31)	63.63 (45.58–81.68)	94.92 (77.61–112.24)
Q2	109.48 (90.19–128.77)	90.89 (79.45–102.33)	134.77 (106.92–162.62)^c^
Q3	125.99 (99.44–152.55)	117.18 (94.94–139.41)	104.02 (73.92–134.13)
Q4	138.24 (112.90–163.59)	124.69 (97.85–151.53)	112.00 (86.26–137.74)
**Diet/light soft drinks**			
Q1	-	1.98 (1.51–2.44)	0.53 (0.29–0.77)^c^
Q2	-	2.10 (1.60–2.60)	0.63 (0.38–0.89)^c^
Q3	-	2.71 (1.85–3.58)	1.25 (0.68–1.82)^c^
Q4	-	5.37 (2.96–7.77)	1.92 (0.84–2.99)
**Other drinks (diet/light and regular**)^b^			
Q1	5.81 (4.11–7.51)	6.29 (5.35–7.26)	6.22 (5.44–7.00)
Q2	7.00 (5.29–8.71)	8.33 (7.28–9.38)	10.06 (9.18–10.95)^d^
Q3	6.55 (4.13–8.96)	9.30 (7.56–11.04)	14.49 (12.82–16.17)^c^^,^ ^d^
Q4	14.18 (10.14–18.22)	16.96 (13.12–20.80)	19.55 (16.58–22.51)

Brazilian Household Budget Surveys, 2002**–**2003; 2008**–**2009; 2017**–**2018.

a Mean based on linear regression models the expected values for income control (values predicted by model);

b in 2002**–**2003, included dairy drinks without yogurt; regular artificial juices or teas; regular and diet/light soymilk powder; in 2008**–**2009 and 2017**–**2018 included dairy drinks without yogurt; regular artificial juices or teas; regular and diet/light soymilk powder; diet/light yogurts; diet/light milk-based drinks; and diet/light artificial juices and teas;

c significant difference compared to the previous year, based on lack of overlap between the intervals;

d significant difference between the years: 2002**–**2003 and 2017**–**2018, based on lack of overlap between the intervals.

## Discussion

The results of the analysis of representative data on Brazilian households revealed a decrease in the acquisition of minimally processed beverages and the stability of ultra-processed beverages over the years. Considering simultaneously the evolution of minimally processed and ultra-processed beverages is relevant to assess a possible replacement behavior, which seemed to happen among lower income level households. In the period analyzed, considering the beverage subgroups, there was a decrease in the purchase of whole and skimmed milk and an increase in the purchase of other ultra-processed drinks. Milk was the most purchased beverage in volume for home consumption in 2002–2003, while regular soft drinks were the most purchased in 2017–2018. Also, there is a greater purchase of regular soft drinks, diet/light soft drinks, and other ultra-processed drinks among households with higher consumption of UPF (fourth quartile), when compared to those with lower consumption (first quartile).

In Brazil, beverages represented 17.1% of total daily energy intake (27). Despite the general stability in the acquisition of ultra-processed beverages verified in the present study, especially considering regular soft drinks, data from the Surveillance of Risk Factors by Telephone Survey (VIGITEL), which evaluates individuals residing in Brazilian capitals and the Federal District, pointed to a reduction in regular consumption (5 days/week or more) and the volume of sweetened beverages consumed (considering soft drinks and artificial juices plus their artificially sweetened versions), with emphasis on sugary drinks, between 2007 and 2016 (21). Despite this reduction in the capital, the same behavior does not seem to have happened in the whole country. Besides the likely different behavior according to the residence locations, one hypothesis for our result is related to possible changes in beverage portion size over the years (28). According to a report by the Pan American Health Organization, between 2000 and 2013, the sales volume of ultra-processed beverages in Brazil had an annual increase of 2.1% from 69.5 to 90.9 kg/year. The average annual increase in Latin America was lower, representing 1.8%, being greatest in Bolivia, Peru, Chile, and the Dominican Republic (29).

Evidence shows that ultra-processed foods, especially sugary drinks, are the main foods consumed out-of-home (30, 31). This study carried out analyses based on food items purchased for consumption at home, which despite not representing the totality of food consumption, continue to be important, as the consumption outside the home is decreasing (30). Therefore, to some extent, our values are underestimated, and the scenario could be more critical.

In addition to the harmful health effects associated with UPF, such as the higher occurrence and/or incidence of obesity, high blood pressure, cancers, and other non-communicable diseases (NCDs) (32–40), the high intake of artificial juices and soft drinks, rich in added sugar, is associated with weight gain and the metabolic syndrome (6, 7, 41, 42), type 2 diabetes (8), arterial hypertension (9), and obesity-related cancers (4, 10). Whereas artificially sweetened beverages are more associated with obesity, intestinal dysbiosis and cancers are not related to obesity (7, 11). Furthermore, the consumption of ultra-processed beverages in general, sugary or artificially sweetened, is associated with a higher incidence of arterial hypertension (43). The findings of this study are worrying, because with the increasing trend in the purchase of other ultra-processed drinks and the high consumption of soft drinks (regular and diet/light) by the population with greater consumption of ultra-processed foods, the risk of developing NCDs—which are considered the most expensive diseases in the world (44)—can be potentiated.

Considering the above and our results, the relevance of the Brazilian Strategic Actions Plan for Tackling Chronic Non-Communicable Diseases 2021–2030 (23) is reinforced to include a goal related to reducing the consumption of sweetened beverages and monitoring the country about this goal by carrying out national representative dietary surveys, such as Household Budget Surveys.

In this sense, effective regulatory public policies related to sweetened beverages should be implemented to reduce their consumption in Brazil. One of the central measures to be taken is the taxation of ultra-processed beverages, especially sugary drinks. Latin American countries such as Mexico and Chile showed satisfactory results in terms of reduced consumption due to the taxation of these beverages (45–47). The regulation of the offering of ultra-processed beverages in schools is also an important axis of the agenda to promote adequate healthy food that needs progress in Brazil (48). Furthermore, considering the problems related to the consumption of ultra-processed foods and beverages, front-of-package warning labeling that simply and informs the quality of food items is of great importance, as it can contribute to the decision-making of individuals (49, 50). In Brazil, a frontal nutrition labeling model was approved, which come into force in October 2022 (51). Despite being advance, this model is not considered the most efficient (52), and still, it does not warn about the use of additives and sweeteners, which are often used in diet/light versions of ultra-processed beverages (53). Additionally, considering the stratification of different populational groups in the monitoring is also relevant since it allows the identification of strategic contexts to reinforce the interventions and public health policies. According to our results, the worst scenario was verified for the most vulnerable households, where the replacement of minimally processed beverages by ultra-processed beverages seemed to be more intense.

The present study has some limitations. Household food purchase data do not allow stratification of the analyses according to individual variables, such as sex and age, which influence food consumption and diet quality, but we included analysis related to income, another variable related to food consumption (54). Food consumption that occurs outside the home is not detailed enough to be included in the analyses and the consumption of ultra-processed foods tends to be higher in this food context (31). However, food consumption outside the home is decreasing in Brazil (30) and household purchase data provide a good prediction of individual consumption (55, 56) and provide information related to all individuals in the household, different from individual consumption data, which assess only those older than 10 years (20).

As strengths of the study, we highlight the innovation in evaluating the trend in the volume of non-alcoholic beverages purchased for consumption at home, categorized according to the NOVA classification (1), which is recognized by international agencies (29, 57) and is adopted in Brazilian Dietary Guidelines (58), and including ultra-processed beverages in their diet/light versions, as these are often “hidden” in consumption analyses because they have low or no energy. Complementing previous analyses related to the frequency of consumption is important to contribute to the monitoring, offering more elements to understand the scenario of consumption.

In conclusion, the results of the analysis of representative data on Brazilian households revealed that the volume of minimally processed beverages purchased declined, while ultra-processed beverages remained stable over 15 years. The acquisition of both minimally processed and ultra-processed beverages was influenced by income with the intensity of changes differing among income groups. The monitoring of beverage consumption by national representative surveys is important and effective public policies regulating these beverages should be implemented to reduce the consumption of ultra-processed beverages, those include taxation, restricted access to these drinks in settings such as schools, and clear nutritional labeling.

## Data availability statement

Publicly available datasets were analyzed in this study. This data can be found here: https://www.ibge.gov.br/estatisticas/sociais/populacao/24786-pesquisa-de-orcamentos-familiares-2.html?=&t%25252520=what-is&t=microdados.

## Ethics statement

Ethical review and approval was not required for the study on human participants in accordance with the local legislation and institutional requirements. Written informed consent for participation was not required for this study in accordance with the national legislation and the institutional requirements.

## Author contributions

NO contributed to the conception, design, data interpretation, performed all statistical analyses, drafted, and critically revised the manuscript. DC contributed to the conception, design, data interpretation, drafted, and critically revised the manuscript. Both authors revised the final version of the manuscript, gave their final approval, and agreed to be accountable for all aspects of the work.

## Funding

This study was supported by the following Brazilian research agencies: Coordination for the Improvement of Higher Education Personnel, Brazil (CAPES) (financing code 001 and process number 88881.637476/2021-01) and Carlos Chagas Filho Foundation for Supporting Research in the State of Rio de Janeiro (FAPERJ) (process numbers: E-26-010.100930/2018, E-26/202.667/2018, and E-26/210.064/2021).

## Conflict of interest

The authors declare that the research was conducted in the absence of any commercial or financial relationships that could be construed as a potential conflict of interest.

## Publisher's note

All claims expressed in this article are solely those of the authors and do not necessarily represent those of their affiliated organizations, or those of the publisher, the editors and the reviewers. Any product that may be evaluated in this article, or claim that may be made by its manufacturer, is not guaranteed or endorsed by the publisher.
